# Use of Chloroform in Ophthalmic Surgery

**Published:** 1870-09

**Authors:** H. W. Boyd

**Affiliations:** Professor of Anatomy, Chicago Medical College


ARTICLE XXVII.

### USE OF CHLOROFORM IN OPHTHALMIC SURGERY.

By H. W. BOYD, Professor of Anatomy, Chicago Medical College.

The discoveries of Chloroform and Ether are considered among the greatest achievements of modern science, and their administration as anæsthetics for the alleviation of pain in surgical operations has become a world-wide practice. There are many cases which, in all probability, would never be cured, were it not for the fact that the patient could be made insensible to pain during the performance of a surgical operation necessary for the cure of the disease. It has become the practice of many surgeons to administer anæsthetics indiscriminately to all classes of cases, whether the operation is attended with much pain or little. This, I think, is wrong; for there is a class of operations in which the use of anæsthetics should be refrained from, save in exceptional cases, where the patient is irritable, restless, without self-control, and refuses to be operated on unless he has chloroform or ether. The indiscriminate use of anæsthetics has, no doubt, been a prolific source of mischief; doubtless many cases of death from chloroform might have been avoided had a little discriminating care been exercised in considering the character and constitution of the patient previous to its administration. Persons have died from chloroform where the operation was so trivial that no anæsthetic was really required, such as extracting a tooth. But the class of cases to which I would allude as not requiring anæsthetics are those included under the head of Ophthalmic Surgery. There are but few operations connected with the eye in which they should ever be used. There is a popular and correct feeling that the eye is an exceedingly delicate organ, and yet the eye is not so highly sensitive as to give rise to much pain in many of the operations performed on it. Take, for example, the operation for extraction of cataract; the pain attending it is not severe,—in fact, it is only trivial; and yet it is the custom of of most surgeons to administer an anæsthetic for this operation. After some observation on this point, I am satisfied that the percentage of success is greater in the hands of those who operate without, than it is with those who always use them. Nearly every operator of extended practice can point to cases of failure in his own experience, which he lays to the use of chloroform or ether. In this operation, aside from the risk of death from the chloroform, it is of material advantage for the surgeon to have the co-operation of the patient, as this will facilitate the exit of the cataract, and at the same time we have none of the risks from the dangerous excitement or restlessness and vomiting caused by the anæsthetic; and we also avoid the nervous prostration which is liable to derange the appetite and affect the functions of nutrition and digestion, which may have very material effect on the healing process, especially in persons somewhat advanced in life, to which class a large majority of cataract patients belong.

The operation for iridectomy is attended with but little pain, and may be better performed without than with anæsthetics. The same remark applies to staphyloma or entropion. In the operation for strabismus, not much can be accomplished with any degree of certainty, unless the patient co-operates with the surgeon, which he is not capable of doing if he is under the influence of an anæsthetic. In this operation, I believe, much mischief has been accomplished by operating while the patient is under the influence of an anæsthetic. Under such circumstances it is impossible for the surgeon to tell when he has divided a sufficient amount of tissue, and he is liable to divide too much, and cause the visual axes to diverge; or, being a careful, prudent, operator, he may fail to divide quite enough and allow a certain amount of convergence or divergence (according as the case is one of convergent or divergent strabismus) to remain; and, in such an event, he must either administer more of the anæsthetic and operate again, or he must postpone until some future time and perform another operation.

And aside from all this unpleasantness, the fuss and struggle sometimes experienced in getting the patient under the influence of the anæsthetic, and the vomiting and disagreeable sickness resulting from it, really causes the patient more suffering than the pain attendant upon the operation. A little resolution and fortitude on the part of the patient will be of infinitely greater service to both patient and surgeon than any amount of anæsthetics. This is true of nearly all operations on the eye, except, perhaps, enucleation of the eye-ball, or some plastic operation on the lids. I do not wish to be understood as undervaluing anæsthetics, for no one can have a more exalted appreciation of their value than myself. In amputations, resections, large tumors, or small but deeply-seated tumors, plastic operations, and operations where extensive dissecting is required, and, in fact, nearly all the operations of general surgery, it would seem now impossible to get along without anæsthetics. But in operations where the pain is so slight that the patient does not suffer materially, and where it is of great advantage to have the co-operation of the patient, then the administration of anæsthetics should be dispensed with, save when the patient has no resolution or self-control, and will not submit to the operation without it.

